# Visible light high-resolution imaging system for large aperture telescope by liquid crystal adaptive optics with phase diversity technique

**DOI:** 10.1038/s41598-017-09595-2

**Published:** 2017-08-30

**Authors:** Zihao Xu, Chengliang Yang, Peiguang Zhang, Xingyun Zhang, Zhaoliang Cao, Quanquan Mu, Qiang Sun, Li Xuan

**Affiliations:** 1 State Key Laboratory of Applied Optics, Changchun Institute of Optics, Fine Mechanics and Physics, Chinese academy of Sciences, Changchun, Jilin 130033 China; 2 Graduate School of the Chinese Academy of Sciences, Beijing, 100039 China; 3 Changchun Institute of Optics, Fine Mechanics and Physics, Chinese Academy of Sciences, Changchun, Jilin 130033 China

## Abstract

There are more than eight large aperture telescopes (larger than eight meters) equipped with adaptive optics system in the world until now. Due to the limitations such as the difficulties of increasing actuator number of deformable mirror, most of them work in the infrared waveband. A novel two-step high-resolution optical imaging approach is proposed by applying phase diversity (PD) technique to the open-loop liquid crystal adaptive optics system (LC AOS) for visible light high-resolution adaptive imaging. Considering the traditional PD is not suitable for LC AOS, the novel PD strategy is proposed which can reduce the wavefront estimating error caused by non-modulated light generated by liquid crystal spatial light modulator (LC SLM) and make the residual distortions after open-loop correction to be smaller. Moreover, the LC SLM can introduce any aberration which realizes the free selection of phase diversity. The estimating errors are greatly reduced in both simulations and experiments. The resolution of the reconstructed image is greatly improved on both subjective visual effect and the highest discernible space resolution. Such technique can be widely used in large aperture telescopes for astronomical observations such as terrestrial planets, quasars and also can be used in other applications related to wavefront correction.

## Introduction

PD technique was firstly proposed by Gonsalves in 1979. After theoretical development and improvement of years, now it plays a significant role in the field of adaptive optics^1, 2^. The goals of PD are reconstructing high-resolution images and estimating phase aberrations simultaneously by collecting two or more degraded images of original object. In PD theory, specific and known phase diversities are introduced among images, such as defocus, astigmatism, coma, etc. As an advanced wavefront sensing technology, compared with traditional wavefront sensing method, PD has many advantages. For example, its optical layout is not complex. Also, it is suitable to both point sources and extended objects. Also, it can achieve high-resolution images without wavefront correcting through optical devices^3, 4^. In 1994, PD technique was applied on solar imaging system successfully^5, 6^. It improved the resolution of image effectively by eliminating the influence of atmospheric turbulences. In 1996, R.A.Carreras *et al*. employed PD technique for the optical imaging of the binary star *μ* Scorpio with a separation of 1.1 arc seconds on the 81 centimeter Beam Director Telescope (BDT)^7^. In 1997, PD was introduced successfully to the piston error detection on segmented mirrors of the prime mirror of Keck II telescope^8^. In 2008, Mugnier *et al*. proposed long-exposure PD technique which let PD technique can be used for the long exposure adaptive optics corrected images for quasi-static aberrations sensing and high-resolution image reconstructing^9^.

In order to detect the dark-weak objects, large aperture telescope has been universally accepted as an effective way. There are more than eight large aperture telescopes (larger than eight meters) which are equipped with adaptive optic systems in the world until now. However, the deformable mirror adaptive optics (DM AO) has its own limitations such as the difficulties of increasing actuator number of deformable mirror, so most of the deformable mirror adaptive optics systems (DM AOS) on large telescope work in the infrared wave band. As a result, liquid crystal adaptive optics (LC AO) has attracted many attentions. LC AO has been deeply studied and achieved a series of developments^10–16^. Air Force Research Laboratory introduced dual-frequency liquid crystal to astronomical adaptive optics system and applied it on the AEOS 3.67 meter telescope^17^. Zhaoliang Cao *et al*. performed an open-loop liquid crystal adaptive optics correction (LC AOC) experiment on a 1.2-meter telescope. A star with a visual magnitude of 4.45 was corrected and the 0.31″ angular resolution was achieved^18^. Meanwhile, LC AO has also been widely applied to other fields, such as correction of the aberrations in the human eye^19^, beam steering^20^ and optical testing^21^. However, the images after open-loop LC AOS are still affected by the residual aberrations due to the slow response speed of liquid crystal and the open-loop strategy. The resolution improvement of images after LC AOS is still required, but the traditional PD technique using beam splitter is not suitable for LC AOS due to the non-modulated stray light from LC SLM.

LC SLM, which can generate various kinds of modes of wavefront aberrations, is playing an increasing important role in PD. Norihide Miyamura used the LC SLM as a phase diversity generator to describe the optimal phase diversity selection in PD, and then demonstrated its validation by laboratory test^22, 23^. In this paper, we apply LC SLM into PD technique and propose a two-step high-resolution optical imaging approach containing open-loop LC AO and PD. Firstly, open-loop LC AOS is applied to make a compensation to most of the phase aberrations caused by atmosphere turbulences. After that, PD is used to estimate the residual phase aberrations and further eliminate the residual blur on the focused images. Inside the approach, a novel PD strategy is proposed which adds known phase diversity to the open-loop correction signal by LC SLM to realize open-loop adaptive optical correcting and generation of phase diversity of PD meanwhile. Compared to the traditional PD, the novel PD strategy can overcome the problem of non-modulated stray light. And it also can introduce any phase aberration into the imaging branch to realize free selection of phase diversity functions. The approach is firstly studied by numerical simulations, and then demonstrated by experiments on the LC AOS. Results show that, the LC AOS with PD technique is available no matter in large or small turbulences and the reconstructed images are obviously better than the focused image after open-loop correction. Such technique can be widely used in large aperture telescopes for astronomical observation such as the studies for terrestrial planets, quasars and other applications related to wavefront correction.

## Results

### Novel PD strategy on LC AOS

In the open-loop LC AOS, the wavefront sensor (WFS) directly measures the wavefront distortions caused by atmosphere turbulences, and the liquid crystal wavefront corrector (LC WFC) compensates for the distorted wavefront in real time. However, the open-loop LC AOS has the disadvantages of slow response speed of liquid crystal and open-loop strategy which cannot detect the residual aberrations. Due to these disadvantages, the compensation to the wavefront distortions cannot be perfect and the resolution of images cannot reach the diffraction limitation. There remains residual blur on the focused image after open-loop LC AOC. In order to eliminate the residual blur and improve the quality of image, we introduce PD technique to the open-loop LC AOS and establish a two-step high-resolution optical imaging approach. First of all, open-loop LC AO technology is used to correct the large proportion of the wavefront distortions produced by atmosphere turbulences. Then PD will be used to estimate the residual aberrations and remove the blur on the collected images.

The traditional PD technique, which uses a beam splitter to split the light for the collection of the focused image and the defocused image in DM AOS^24, 25^, cannot be used directly in LC AOS because of the existence of non-modulated stray light generated by LC-SLM. As a result, a novel PD strategy for LC AOS is proposed. The light degraded by atmosphere turbulences enters into an open-loop LC AOS. The wave band from 400 nm to 700 nm is reflected into the wavefront sensing branch, and the unknown turbulences will be detected by Shack-Hartmann wavefront sensor (SH-WFS). The wave band from 700 nm to 900 nm is transmitted into the wavefront correcting branch. A polarization beam splitter (PBS) is used to divide the beam into two bundles polarized perpendicular to each other. The separated beams enter into two LC SLMs for wavefront correcting, meanwhile, one of the LC SLMs generates the phase diversity function.

Compared to the traditional PD technique, the biggest difference in proposed PD strategy above is that one LC SLM does the correction work normally, while the other LC SLM introduces an arbitrary phase diversity function together with the normal correction signal. The main advantages of new PD strategy for LC AOS are that the problem of non-modulated stray light is solved (numerical simulation section) and real-time free phase diversity function selection is achieved.

### Numerical simulations

Due to the imperfect alignment of the liquid crystal molecules of LC SLM, a small fraction of light polarized randomly will be generated after LC SLM called zero-order stray light^26^. The zero-order stray light cannot be modulated by the LC SLM and brings bad influence to wavefront estimating. The degraded images of PD include both first-order diffracted light which is modulated entirely and zero-order light that cannot be modulated by LC SLM. For the proposed PD strategy, the phase diversity function is introduced by the LC SLM. The focused image consists of the zero-order light which is degraded by turbulence aberrations and the first-order light that is degraded by residual aberrations after correction. The diversity image is composed of the first-order light which is degraded by both the residual aberrations and known phase diversity function (here we use defocus as phase diversity function.), but the zero-order light is still degraded by the same turbulence aberrations as in the focused image (because the zero-order light cannot be modulated by the LC SLM, phase diversity function cannot be applied onto the zero-order light). As described in the following formulas:

1 $$i=FO{L}_{{\phi }_{r}}+ZO{L}_{\phi },$$

2 $${i}_{d}=FO{L}_{{\phi }_{r}+\theta }+ZO{L}_{\phi },$$

3 $${i}_{d\_traditional}=FO{L}_{{\phi }_{r}+\theta }+ZO{L}_{\phi +\theta },$$

where *i* and *i*_*d*_ represent the focused image and the diversity image. *FOL* and *ZOL* represent the first-order light and the zero-order light, respectively; *φ* is the turbulence aberrations; *φ*_*r*_ is the residual aberrations to be estimated; and *θ* is the known phase diversity function. The subscript indicates the degrading effect. From equations (1–2) we can see that the zero-order light does not satisfy the PD relations which can be treated as simple intensity noise in the new strategy. But for the traditional PD strategy with prism, zero-order light in the diversity image is degraded by both turbulence aberrations and the known phase diversity function, that is to say the zero-order light also satisfy the PD relations and will influence the whole PD process. Above mentioned is the prime difference between the proposed PD strategy in this work and the traditional PD strategy.

In order to analyze the influence of zero-order stray light to aberrations estimation, numerical simulations are performed. We use two random turbulence aberrations to simulate the atmospheric turbulence, a larger one and a smaller one respectively. The forms of two wavefronts are the same and the root-mean-square (RMS) scales are 1.3 *λ* and 0.78 *λ* for the larger and smaller turbulences respectively (here we set *λ* to be 532 nm). As for the residual aberrations to be estimated, we use two random aberrations (aberration a and b) whose RMS scales are 0.17 *λ* and 0.125 *λ* respectively. An optical resolution test board is used as the original object in both the simulations and experiments. To analyze the relationship between the aberrations estimating accuracy and the energy ratio of zero-order light and first-order light, we do the simulations in the range of 1/15 to 1/3 which is the general range of energy ratio of LC SLM. The hybrid particle swarm global optimization algorithm based on the evolutionary particle swarm optimization and the Broyden-Fletcher-Goldfarb-Shanno algorithm for wavefront estimating is used here^27^.

The accuracy of estimated aberrations is described by the ratio of RMSE of aberrations estimation and RMS of aberrations to be estimated. The RMSE is calculated in the following formula:

4 $$RMSE(\hat{\phi })=\sqrt{\frac{\sum _{i=1}^{N}{(\hat{\phi }(i)-\phi (i))}^{2}}{N}},$$

where $\hat{\phi }$ is the estimated aberration, *φ* is the true aberration to be estimated, and *N* represents the number of sampling points in the discrete pupil function.

**Figure 1:**
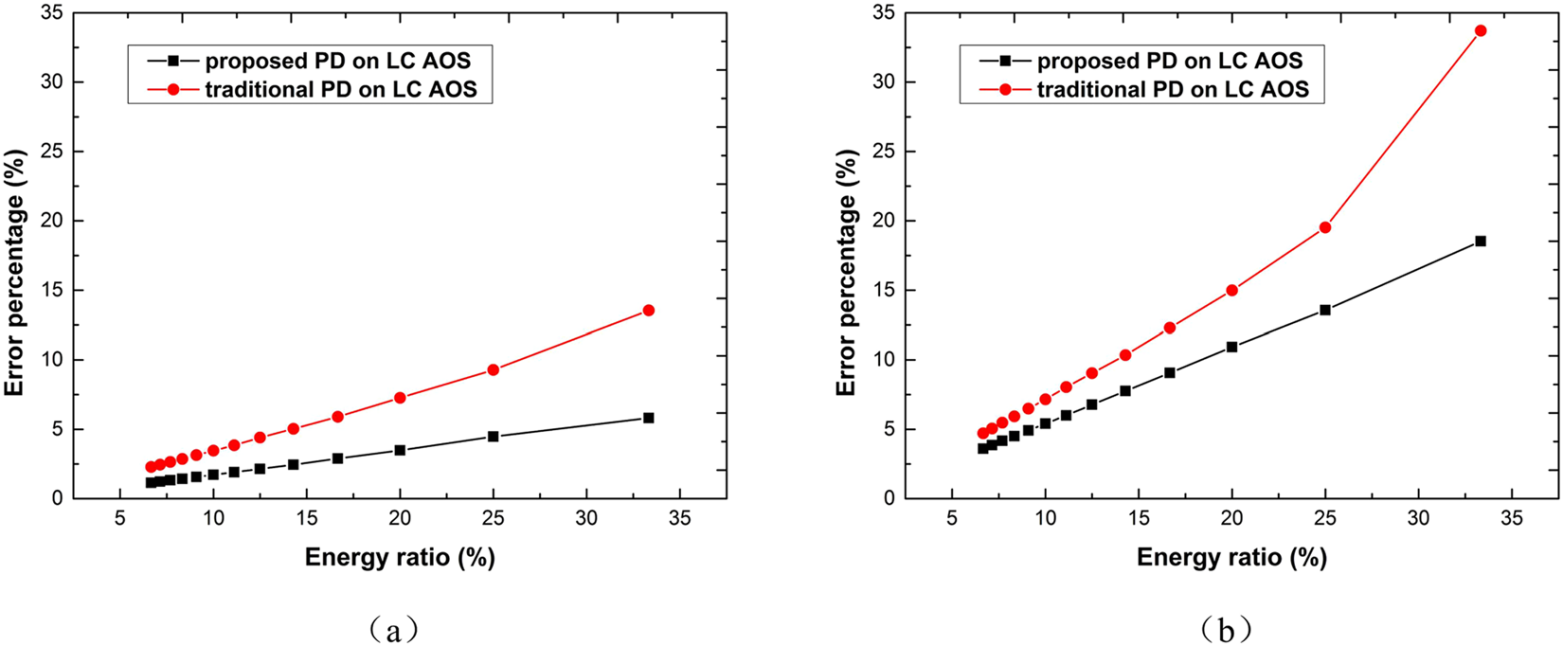
(**a**) Simulation results of the estimating error for larger residual aberration. (**b**) Simulation results of the estimating error for smaller residual aberration b. In both plots, the black lines present the proposed PD strategy and the red lines are for the traditional PD.

Residual aberrations estimation accuracies with the larger turbulence and the smaller turbulence are shown in Fig. 1a and Fig. 1b, respectively. Results show that when the energy ratios of zero-order light and first-order light rise gradually from 1/15 to 1/3, the error percentage of residual aberration estimation increases monotonically. That is to say the accuracy becomes lower with the increasing energy ratio. Keeping the energy ratio within low level is the key point for LC AOS with PD technique. As to the proposed PD strategy in this work, within the simulated energy ratio range, the error percentages are lower than 5% for the larger residual aberration. Compared to the proposed PD strategy, the error percentage of traditional PD is twice as high as the proposed PD within the whole energy ratio range from 1/15 to 1/3. For the smaller residual aberration, the error percentages of proposed PD are no higher than 20%, most of which lower than 10% within the energy ratio range from 1/15 to 1/5. The highest error percentage is 18.52% for energy ratio 1/3, which is much lower than the error of traditional PD 34%. From the results of simulations, we can see that the proposed PD strategy is more accurate than traditional PD strategy, which is very suitable for the estimation of residual aberrations after open-loop LC AO.

### Experiment

**Figure 2:**
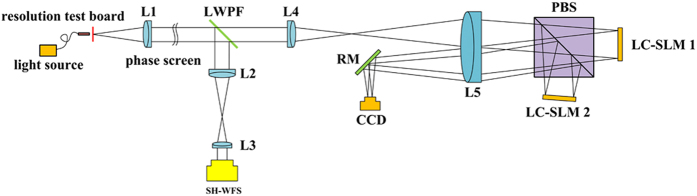
Experimental layout for proposed LC AOS with novel PD strategy.

**Figure 3:**
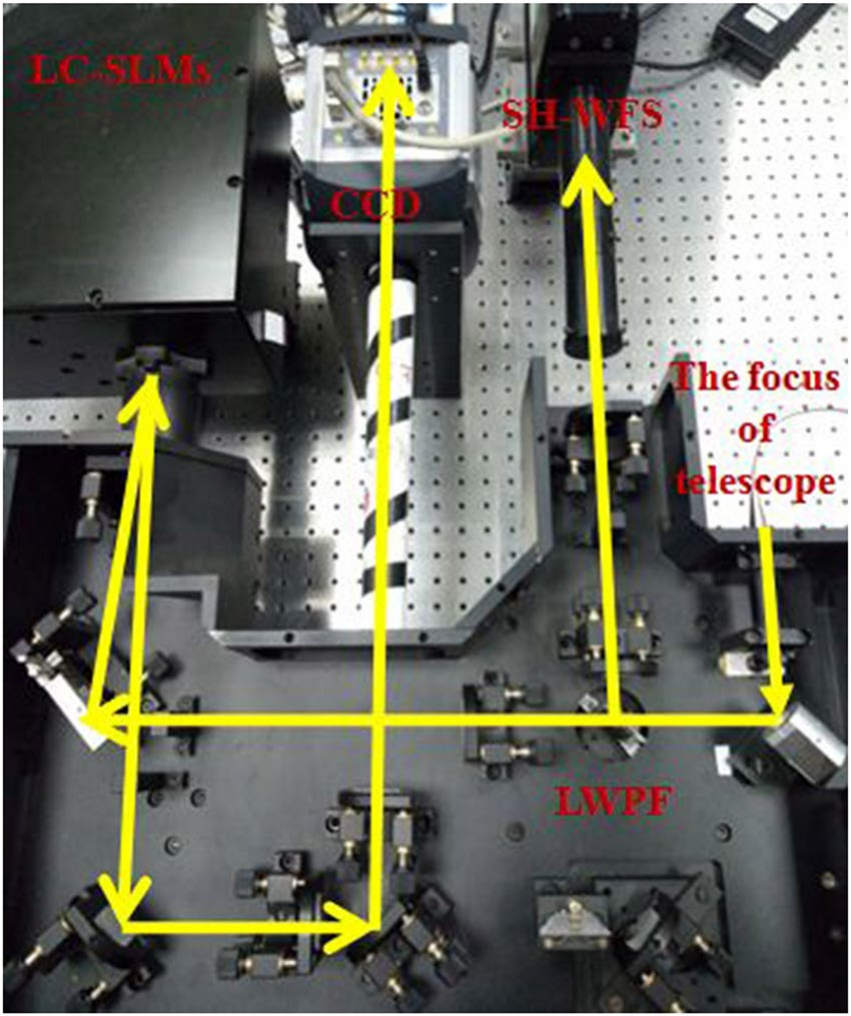
Photo of experimental setup for proposed LC AOS with novel PD strategy.

The performance of the proposed LC AOS with PD technique is studied through experiments, whose experimental setup is shown in Fig. 2. And the photo of experimental setup is also shown in Fig. 3. An optical resolution test board is used as the object which is illuminated uniformly by the optical fiber light source. The transmitted light is collimated by lens L1 and the turbulence phase plate (Lexitek, *r*_0_:1.08 mm) is placed in the path. The short-wave band from 400 nm to 700 nm is reflected by the long-wave passing filter (LWPF) into the wavefront sensing branch. The transmitted light passing through LWPF enters into the wavefront correcting branch. A PBS is used and the S and P polarized components of the light go to two wavefront correctors respectively (LC SLM 1 and LC SLM 2). Finally, the corrected lights enter PD optical layout for residual aberrations estimation and higher-resolution image reconstruction. The focused image and the diversity image will be collected by two areas of the CCD camera located in the focal plane. The 0.5 *λ* PV defocus is introduced by LC SLM 2 as the phase diversity function in this experiment. The LC SLMs used in this work have 256 × 256 pixels, which make them available to six-meter telescopes for 10 cm atmospheric coherent length^28^. Furthermore, owing to the mature TFT technology, LC SLMs with more than 1024 × 1024 pixels are very common, in other words, the LC AOS can be used for telescopes of more than ten meters very easily.

**Figure 4:**
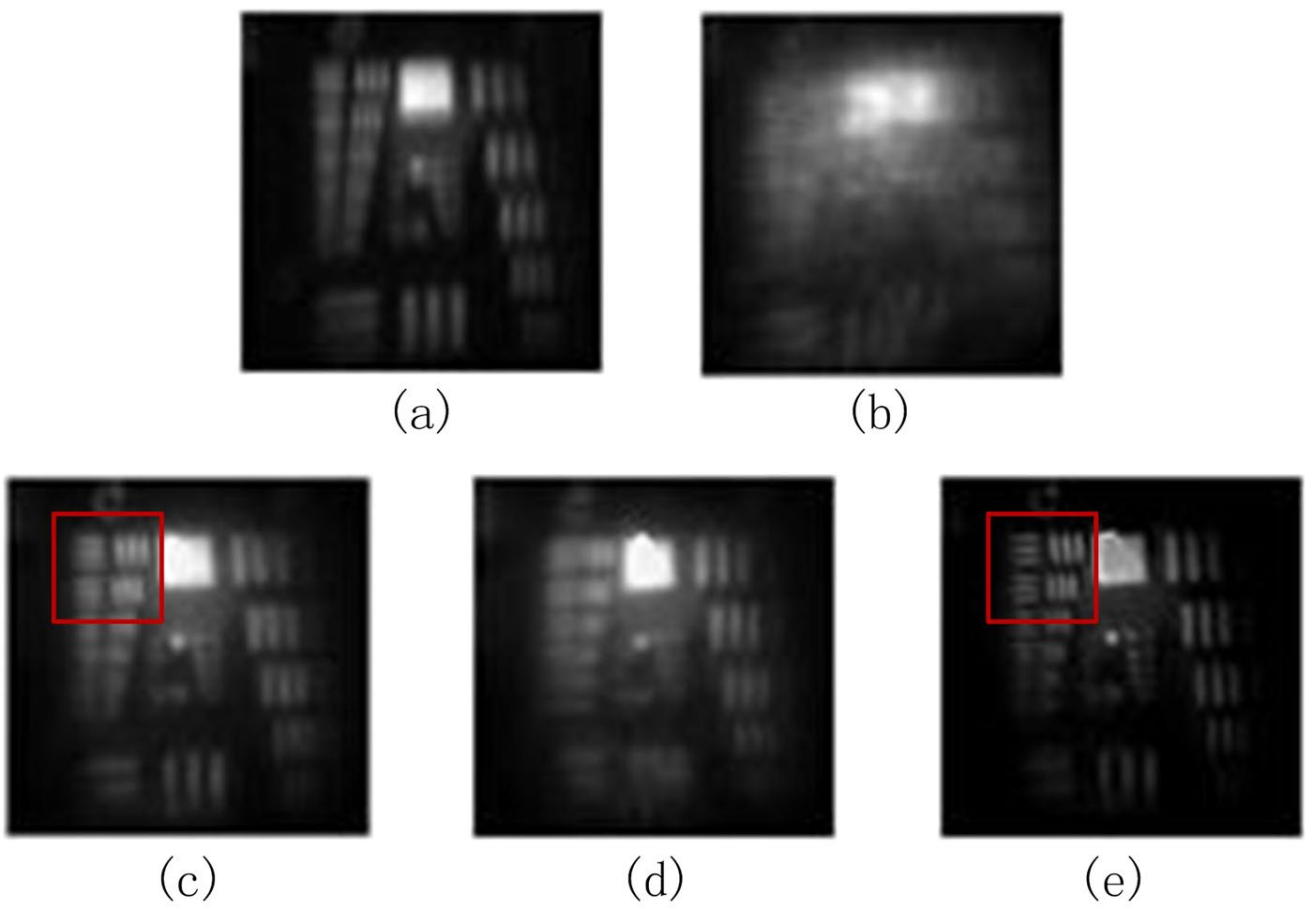
The experimental results of proposed LC AOS with PD technique. (**a**) The undegraded focused image. (**b**) The focused image degraded by phase plate. (**c**) The focused image after open-loop LC AOC. (**d**) The diversity image used in PD technique. (**e**) Image reconstructed by PD technique.

The experimental results are shown in Fig. 4. Figure 4a is the focused image of the system without turbulence aberration to as reference image and Fig. 4b is the focused image degraded by the turbulence phase plate without adaptive correction. The resolution of Fig. 4b is so bad that one cannot recognize any specific details from the image. After open-loop LC AOC, the focused image collected by CCD camera is shown in Fig. 4c. We can see from Fig. 4c, after the compensation to turbulence distortions, the resolution of the image has been improved so much that most of the details in the image can be recognized. The highest space resolution that can be clearly recognized is 20.16 lp/mm, which is equivalent to 1.94× diffraction limitation. However, there still exists residual blur in the image. The edges in the image are still not clear enough and the resolution can be further improved. So the diversity image (see Fig. 4d) is collected simultaneously with the focal plane image to estimate the residual aberrations and reconstruct higher-resolution image. The reconstructed image is shown in Fig. 4e. Compared with Fig. 4c, the reconstructed image by the novel PD strategy is clearer. Residual blur is generally eliminated and the specific details are well presented. The image edges are sharper and the resolution is higher. Especially for the red mark regions on both of the images, an obvious improvement of resolution is presented. The highest space resolution that can be clearly recognized reaches more to 35.92 lp/mm, which is equivalent to 1.08× diffraction limitation.

## Discussion

In this work, a novel PD strategy for LC AOS is proposed. The proposed strategy mainly has two advantages. The first one is that arbitrary phase diversity functions can be introduced in PD estimating in real time due to the LC SLM. Jean *et al*. made an analysis on the optimized selection of phase diversity function based on Cramer-Rao bound theory^29^. The results indicated that in some cases, other phase diversity functions may be more suitable than defocus, although defocus diversity works well in the most of cases. Also, the selecting principle of phase diversity function is much different for the extended object and point object. In this novel PD strategy, LC SLM is used which could introduce arbitrary phase diversity function to achieve the optimization of phase diversity function. Compared to the novel PD strategy proposed in this work, the traditional strategy with beam splitter or a combination of beam splitter and prism can only introduce defocus as the phase diversity function and cannot change in real time.

The second advantage of the proposed PD strategy is that the problem of the zero-order non-modulated stray light from LC SLM can be solved. When the light pass through LC SLM, the zero-order non-modulated stray light will be generated due to the imperfect alignment of LC molecular, no matter for the parallel-aligned nematic LC SLM or the twisted nematic LC SLM. In the traditional PD strategy, both zero-order light and first-order light are modulated by known phase diversity function in the diversity image. Both of them satisfy the phase relation of PD. But the wavefront of zero-order light is not corrected by LC SLM and it will lead to big error of phase estimation in LC AOS. But for the proposed PD strategy, the zero-order light is not modulated by known phase diversity function in the diversity image, so the zero-order light does not satisfy the phase relation of PD which can be treated as a pure intensity noise to the images. So this novel proposed PD technique for LC AOS could enhance the accuracy of residual aberrations estimating after LC AOS.

In summary, a two-step high-resolution optical imaging approach consisting of open-loop LC AOS and PD technique is proposed in this work. Firstly, open-loop LC AOS is used to correct most of the wavefront distortions caused by atmosphere turbulences. And then, PD is used to estimate the residual phase aberrations and remove the residual blur on the collected images. Inside the second step, the proposed PD strategy solves the problem of zero-order non-modulated stray light introduced by LC SLM and reduces the error of residual aberration estimating significantly. The phase diversity function can be selected freely due to the LC-SLM in the novel PD strategy. The proposed approach is studied by both numerical simulations and experiments. In numerical simulations, the results show that the accuracy of proposed PD strategy is much higher than the traditional PD strategy in LC AOS within the energy ratio from 1/15 to 1/3 for zero-order and first order light. In experiments, the proposed approach is successfully applied on a LC AOS and removes the residual blur of the open-loop corrected focused image. The reconstructed image is much better in both visual effect and the highest discernible space resolution. The highest space resolution that can be clearly recognized reaches up to 35.92 lp/mm, which is equivalent to 1.08× diffraction limitation. The proposed approach may have potential applications in various domains, especially for high-resolution adaptive imaging for visible light on telescopes with large aperture.

## Methods

### PD theory

**Figure 5:**
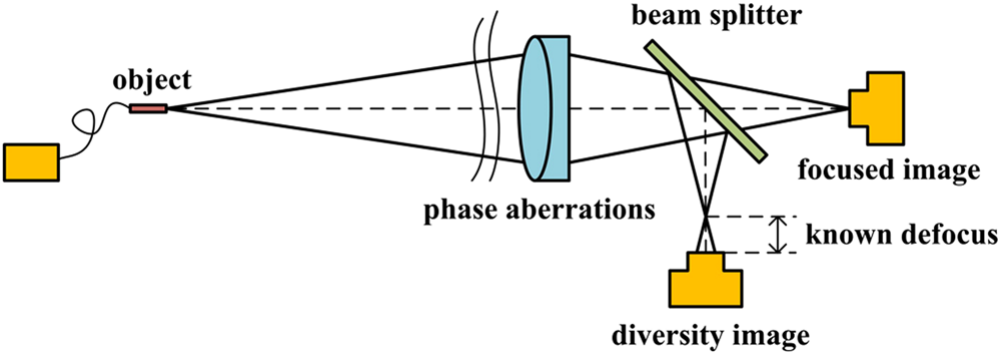
Optical layout of phase diversity technique.

PD technique which employs a series of degraded images related to certain known phase diversities is widely used in simultaneous phase aberration estimation and image reconstruction. This technique can eliminate the non-uniqueness of the solution, which is also the primary difficulty due to the indetermination of relation between the phase distribution in the pupil and the optical transfer function (OTF) of the system^30^. Generally, we use a pair of images one of which is degraded by a known defocus aberration. As shown in Fig. 5, the first image degraded by unknown phase aberration is obtained in the focal plane and the second image further degraded by known defocus aberration is acquired in a specific defocused plane.

The error metric under Gaussian noise can be established through maximum likelihood estimation theory in the following expression:

5 $$E(o;\phi )=\sum _{k=1}^{K}\sum _{x,y}{|{d}_{k}(x,y)-o(x,y)\ast {h}_{k}(x,y)|}^{2},$$

where *o*(*x*, *y*) is the intensity distribution of the observed object; *h*_*k*_(*x*, *y*) is the PSF with diversity *k*; *d*_*k*_(*x*, *y*) is the *k* th diversity image with noise;

Through dimension reducing, the error metric of unknown phase aberrations can be obtained, as well as the observed object expression:^31^

6 $$E(\alpha )=\sum _{u,v}\frac{{|{D}_{1}(u,v){H}_{1}(u,v)-{D}_{2}(u,v){H}_{2}(u,v)|}^{2}}{{|{H}_{1}(u,v)|}^{2}+{|{H}_{2}(u,v)|}^{2}},$$

7 $$o(x,y)=F{T}^{-1}(\frac{{D}_{1}(u,v){H}_{1}^{\ast }(u,v)+{D}_{2}(u,v){H}_{2}^{\ast }(u,v)}{{|{H}_{1}(u,v)|}^{2}+{|{H}_{2}(u,v)|}^{2}}),$$

where *D*_*k*_(*u*, *v*) and *H*_*k*_(*u*, *v*) are the discrete Fourier transforms of *d*_*k*_(*x*, *y*) and *h*_*k*_(*x*, *y*).

Through nonlinear optimization algorithm, the error metric can be minimized with the global seeking of *H*, which is the goal for PD estimating. Then the image can be reconstructed through equation (7).

### Proposed novel PD strategy for LC AOS

The optical layout of the novel two-step high-resolution optical imaging LC AOS with novel PD strategy used in this work is shown in Fig. 2. The unknown turbulence distortions will be detected by SH-WFS and the LC SLMs will make an open-loop wavefront correction for them. As shown in Fig. 2, two beams after open-loop correcting are reflected by a mirror and collected by a CCD camera located in the focal plane of the system to achieve a PD reconstruction. The most important difference between the novel PD strategy and traditional one is that the diversity function is applied by the LC SLM rather than the defocus distance or prism. The LC SLM 1 does the correction work normally, but the LC SLM 2 introduces an arbitrary phase diversity function together with the normal correction signal. The focused image and the diversity image could be collected in the same time by two areas of one CCD camera located in the focal plane.
